# Alveolar-Membrane Diffusing Capacity Limits Performance in Boston Marathon Qualifiers

**DOI:** 10.1371/journal.pone.0044513

**Published:** 2012-09-11

**Authors:** Kaleen M. Lavin, Allison M. Straub, Kathleen A. Uhranowsky, James M. Smoliga, Gerald S. Zavorsky

**Affiliations:** 1 Human Physiology Laboratory, Marywood University, Scranton, Pennsylvania, United States of America; 2 The Commonwealth Medical College, Scranton, Pennsylvania, United States of America; 3 Department of Physical Therapy, High Point University, High Point, North Carolina, United States of America; University of Pittsburgh, United States of America

## Abstract

**Purpose:**

(1) to examine the relation between pulmonary diffusing capacity and marathon finishing time, and (2), to evaluate the accuracy of pulmonary diffusing capacity for nitric oxide (DLNO) in predicting marathon finishing time relative to that of pulmonary diffusing capacity for carbon monoxide (DLCO).

**Methods:**

28 runners [18 males, age = 37 (SD 9) years, body mass = 70 (13) kg, height = 173 (9) cm, percent body fat = 17 (7) %] completed a test battery consisting of measurement of DLNO and DLCO at rest, and a graded exercise test to determine running economy and aerobic capacity prior to the 2011 Steamtown Marathon (Scranton, PA). One to three weeks later, all runners completed the marathon (range: 2∶22:38 to 4∶48:55). Linear regressions determined the relation between finishing time and a variety of anthropometric characteristics, resting lung function variables, and exercise parameters.

**Results:**

In runners meeting Boston Marathon qualification standards, 74% of the variance in marathon finishing time was accounted for by differences in DLNO relative to body surface area (BSA) (SEE = 11.8 min, *p*<0.01); however, the relation between DLNO or DLCO to finishing time was non-significant in the non-qualifiers (*p* = 0.14 to 0.46). Whereas both DLCO and DLNO were predictive of finishing time for all finishers, DLNO showed a stronger relation (r^2^ = 0.30, SEE = 33.4 min, *p*<0.01) compared to DLCO when considering BSA.

**Conclusion:**

DLNO is a performance-limiting factor in only Boston qualifiers. This suggests that alveolar-capillary membrane conductance is a limitation to performance in faster marathoners. Additionally, DLNO/BSA predicts marathon finishing time and aerobic capacity more accurately than DLCO.

## Introduction

In 2010, nearly one half million runners in the United States completed a marathon, representing about 0.2% of the U.S. population over 18 years of age. Many marathoners aspire to qualify for the Boston Marathon, participation in which is restricted to a relatively small percentage of runners by age and gender-graded qualification standards. In 2011, approximately 40,000 runners qualified for the 2012 or 2013 Boston Marathon, representing about 10% of all runners who finished a marathon in the United States. These statistics are readily available to the public online at MarathonGuide.com.

Due to its aura and relative popularity, marathon running has been examined in several scientific studies, with particular attention given to the metabolic [1] and physiological [2], [3], [4], [5], [6] correlates of running a fast marathon. Although these correlates are multifactorial and widely debated [7], it is well established that aerobic capacity (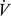
O_2max_) is an important determinant of marathon performance. Approximately 40 to 77% of the variance in marathon performance is attributable to 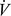
O_2max_ [5], [8], [9], [10], [11]. In addition to aerobic capacity, marathon-specific endurance is related to performance, such that the ability to sustain a higher percentage of 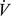
O_2max_ is correlated with a faster marathon [12]. On average, top marathoners (<136 minutes for men, <158 minutes for women) run at 85 to 90% 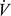
O_2max_ [5], while those in the 156 to 240 minute range run at approximately 75 to 85% of 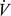
O_2max_ [10], [13].

Aerobic capacity is dependent on the integrated function of major organ systems, including the heart, lung, and skeletal muscle [14]. Unlike the heart and skeletal muscle, the lung does not readily adapt to endurance training [15], [16], possibly limiting 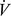
O_2max_ [15]. Even in those who are highly aerobically fit, heavy exercise may cause arterial oxygen pressure to drop ≤80 mm Hg, while the alveolar-to-arterial oxygen pressure difference may increase to ≥25 mmHg [17]. In this way, arterial oxyhemoglobin saturation is reduced, leading to a decrease in 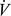
O_2max_ and, subsequently, endurance performance [18], [19], [20].

Recently, alveolar-membrane diffusing capacity (measured at rest) has been shown to be very closely related to 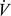
O_2max_ in fit and obese individuals [21], [22] and to longevity in heart disease patients [23]. Specifically, when measured at rest, pulmonary diffusing capacity for nitric oxide (DLNO, mL NO.min^−1^.mmHg^−1^) – a surrogate for alveolar-membrane diffusing capacity for carbon monoxide (DmCO) [24], [25] – has been shown to be related to aerobic capacity in fit men and women, such that for every 1 unit increase in DLNO, 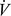
O_2max_ increases by 0.3 mL O_2_. kg^−1^. min^−1^ [21]. The ratio of DLNO to DmCO is debated. It has been said that DmCO  =  DLNO ÷ 2.42, or, more recently, DmCO  =  DLNO ÷ 2.06 to 2.26. As long as the ratio is kept consistent within a study, any percent change in DmCO or pulmonary capillary blood volume is still valid. Furthermore, alveolar membrane conductance is the main pulmonary diffusing capacity component representative of fitness, with the exception of pulmonary capillary blood volume (V_C_) and the blood transfer conductance (Θ) for CO (ΘCO). There are mixed data as to whether DLCO or DLNO is a more valid predictor of aerobic capacity, but overall diffusion capacity does appear to be significantly correlated with aerobic capacity in fit subjects [21], [22]. Nitric oxide (NO) has been shown to bind more strongly than CO to hemoglobin [26], leading to a higher value for membrane conductance and a diffusion measurement more reflective of total membrane diffusion. An additional benefit of measuring DLNO simultaneously with DLCO is a reduction in time and effort of the procedure.

As there is a relation between DLNO and 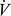
O_2max_, and between 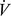
O_2max_ and marathon running performance, it follows that DLNO may be related to marathon performance, such that pulmonary diffusing capacity introduces a limitation that influences marathon performance. However, it is likely that this correlation may be observed in only well-trained marathoners, who are more likely to experience pulmonary limitations to oxygen availability. Pulmonary limitations have been observed in elite athletes, and as many as 50% of highly-trained individuals experience low oxygen concentrations in the blood (hypoxemia), potentially due to diffusion limitation [27]. Whereas hypoxemia is most frequently observed at exercise intensities near maximal exertion, it is possible that endurance events such as a marathon place constraints on the working lung muscles regardless of the submaximal speed at which most runners compete. Indeed, Amann et al. [28] found that pulmonary limitations were capable of significantly decreasing performance in a 5 kilometer cycling time trial. This effect is likely intensified during a marathon, which, although run at a slower pace, is more than eight times as long. Another study shows that seasoned runners experience significant decreases in diffusing capacity following completion of a marathon [29], suggesting an important role for the lungs in an event that requires submaximal speed but maximal overall effort.

Based on these observations, the purpose of this study was to compare the relation between DLNO and DLCO (indexed to body surface area, BSA) and marathon running performance. It was hypothesized that pulmonary diffusion limitation would exist in faster runners, defined herein as those meeting qualification standards for the Boston Marathon (Boston Qualifiers, BQ). Qualification for this prestigious event is dependent on one’s performance relative to age and gender standards, thus eliminating the concerns associated with grouping subjects based on marathon time alone (e.g. creating a younger or predominantly male group). Furthermore, the Steamtown Marathon is a certified qualification course and frequently qualifies over 20% of its participants for the Boston event. This local event therefore provides the opportunity to study differences between sub-elite and more recreational marathoners using a less ambiguous distinction than running pace.

In analyzing these data, the relation between marathon finishing time and DLNO or DLCO in both groups was examined using a linear regression model. No significant relation between these factors was expected in non-qualifiers (non-BQ), whereas qualifiers (BQ) were expected to show a significant correlation between finishing time and DLNO/DLCO. Additionally, it was hypothesized that DLNO would show a stronger relation with marathon finishing time than would DLCO, in agreement with previous findings [21], [30].

## Methods

Twenty-eight endurance trained subjects (18 males, 10 females), reported for preliminary testing 2 to 3 weeks before the 2011 Steamtown Marathon in Scranton, PA. Institutional Review Board-approved informed written consent and a Physical Activity Readiness Questionnaire were obtained from all subjects before participation. Anthropometrics (body mass, height, BSA) and age were obtained, and percent body fat (BF %) was measured using Dual Energy X-ray Absorptiometry (DEXA) (Lunar Prodigy™, GE Medical Systems, Madison, WI).

Pulmonary function tests, consisting of spirometry maneuvers to identify obstructive or restrictive patterns, were conducted according to established guidelines [31]. The maneuver to determine DLNO and DLCO was also performed according to established guidelines [32], with a 5 to 6 second breath-hold [21]. Because this one-step maneuver allows simultaneous measurement of DLNO and DLCO, pulmonary capillary blood volume (V_C_) was then calculated as follows: DmCO was computed as DLNO ÷ 2.42. The 1/ΘCO was determined from Roughton and Forster [33] as (0.73+0.058 · P_A_O_2_) · (14.6/[Hb]), where alveolar oxygen pressure (P_A_O_2_) was 100 mmHg, and the hemoglobin concentration [Hb] was set as 14.6 g. dL^−1^ for males, and 13.4 g. dL^−1^ for females [32]. As such, 1/ΘCO was 1.310 for males and 1.427 for females. V_C_ was then obtained by solving for it using the following equation [33]:



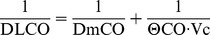



Reference equations were then used to compare each marathon runner’s lung function to normative data from the standard population [34], [35].

After the lung function tests were completed, running economy testing was performed on treadmill at a 2% grade to simulate outdoor running conditions at three different sub-maximal running speeds, each lasting 5 minutes. The treadmill was calibrated before the first subject was tested. Throughout testing, heart rate (HR) was measured using a Polar heart rate monitor (Model S610, Polar Electro USA, Lake Success, NY). Metabolic data were collected using breath-by-breath analysis (Sensormedics Vmax 229D, Viasys, CA).

Assuming that steady state exercise had been achieved within the first three minutes, means for metabolic data for the last two minutes of each stage were computed. Running economy for each stage was computed as the 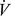
O_2_ required to travel one kilometer and expressed as mL O_2_.min^−1^.km^−1^. Mean running economy for the three speeds was then computed to serve as a measure of overall running economy.

The final stage of the running economy test protocol was followed immediately by a graded exercise test, in which treadmill speed was increased by 0.5 mph every minute until volitional exhaustion. Several cardiorespiratory parameters, including maximum respiratory exchange ratio (RER_max_), maximum heart rate (HR_max_), maximum expired ventilation (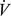
E_max_), and 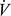
O_2max_ were measured, and the treadmill speed at which 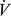
O_2max_ was obtained was recorded (v
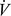
O_2max_). If this speed was sustainable for less than a full minute, the highest speed sustained for 60 seconds was also recorded. Marathon-specific endurance (%_V
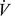
_O_2max_) was calculated by dividing the runner’s mean speed for the Steamtown marathon by v
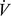
O_2max_, with higher values indicating performance at a greater relative physiologic intensity. Since 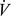
O_2_ could not be directly measured during the race, average 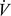
O_2_ for the marathon was calculated from the slope of the regression line between speed and 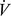
O_2_ for each subject, using the three speeds of the running economy test. Average speed for the marathon was then entered into each subject’s own equation to solve for oxygen consumption.

Univariate ANCOVAs or independent *t*-tests (with a confidence interval of 95%) were used to determine whether differences in finishing time, 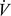
O_2max_ (both absolute, in L.min^−1^, and relative, in mL.kg^−1^.min^−1^), and lung function parameters existed between BQ and non-BQ groups. Age and gender served as covariates for the ANCOVA. Step-wise multiple linear regressions were conducted to determine variables most closely related to finishing time for the entire sample, as well as for each group separately. DLNO and DLCO normalized to body surface area (BSA) were included in regression analyses to account for the effects of body mass and height on lung size. Other predictor variables entered into regression analysis include gender, age, body mass, body fat percentage, 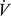
O_2max_ (L O_2_. min^−1^), running economy (mL O_2_. kg^−1^. km^−1^), DLNO, DLCO, and marathon specific endurance. To address the significance of diffusing capacity, the relation between DLNO or DLCO and finishing time was further explored in a bivariate regression analysis for both BQ and non-BQ groups. The data were analyzed by SPSS Version 19.0, (SPSS Inc., Chicago, IL). Statistical significance was declared when *p*<0.05.

## Results

A total of 392 Steamtown Marathon finishers (22% of marathon participants) met qualification standards for the 2012 or 2013 Boston Marathon. Of the 28 subjects in this study, 10 runners (6 males, 4 females) (36%) qualified for Boston. Anthropometric measurements (Table 1) indicated that significant differences exist between BQ’s and non-BQ’s with respect to age and body fat percentage (*p*<0.05).

**Table 1:** Anthropometric Data for Boston Qualifiers and non-Qualifiers.

	BQ (n = 10)	Non-BQ (n = 18)	Total (n = 28)
ANTHROPOMETRICS			
Age (yr)*	33 (9)	40 (7)	37 (9)
	22–50	29–52	22–52
Weight (kg)	64.1 (11.1)	73.2(13.3)	69.9 (13.2)
	42.2–84.5	45.0–103.0	42.2–103.0
Height (cm)	170.7 (9.0)	174.0 (9.2)	172.8 (9.1)
	154.0–186.7	156.0–187.0	154.0–187.0
Body Fat (%)*	13.1 (7.12)	19.1 (6.7)	17.0 (7.3)
	5.1–24.1	9.0–34.5	5.1–34.5
CARDIOPULMONARY VARIABLES AT MAXIMAL EXERCISE			
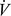 O_2max_ (L/min)	3.75 (0.64)	3.56 (0.64)	3.63 (0.64)
	2.71–4.75	2.25–4.52	2.25–4.75
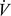 O_2max_ (mL/kg/min)*	59.4 (8.3)	48.7 (5.0)	52.5 (8.1)
	49.1–73.1	38.3–60.4	38.3–73.1
RERmax	1.17 (0.07)	1.16 (0.06)	1.16 (0.06)
	1.06–1.30	1.06–1.28	1.06–1.30
VEmax (L/min)	119.91 (21.10)	112.03 (18.46)	114.85 (19.43)
	82.6–141.13	81.00–149.30	81.00–149.30
HRmax (bpm)	187 (12)	178 (12)	181 (12)
	169–202	152–198	152–202
RUNNING PERFORMANCE			
Running Economy (mL/kg/km)‡	194.5 (13.0)	205.8 (19.1)	202.0 (18.1)
	180.8–224.5	172.9–247.6	172.9–247.6
Running Economy (mL/kg/min)	43.2 (5.9)	36.3 (3.7)	38.8 (5.6)
	36.0–52.0	30.0–44.2	30.0–52.0
Maximum Treadmill Speed for 60 seconds (m/min)*	316 (34)	266 (31)	284 (40)
	271–362	228–316	228–362
Marathon Finishing Time (min)	180.0 (23.1)	242.2 (28.3)	220.0 (40.0)
	142.0–203.0	200.4–289.0	142.0–289.0
% 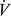 O_2max_ for Marathon	76.4 (6.9)	74.8 (7.2)	75.3 (7.0)
	63.0–82.4	63.0–88.3	63.0–88.3
Specific Endurance (% 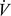 _VO2max_)*	75.31 (5.44)	66.47 (3.97)	69.62 (6.20)
	63.16–81.72	56.26–72.04	56.26–81.72

Data are reported as mean (SD) values and range.

* denotes significant difference (p<0.05) between BQ and non-BQ subjects. Controlling for age and gender using an ANCOVA did not affect the outcome of statistical analyses.

‡ Running Economy calculated at average speed for group; BQ = 222.2 (30.0) m/min, non-BQ = 177.2 (20.4) m/min.

The graded exercise test to volitional exhaustion lasted 10.1(1.2) minutes including the final 5-min running economy bout. The 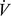
O_2max_ in L O_2_. min^−1^ from that test was not different between groups; however, relative 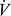
O_2max_ (mL O_2_. kg^−1^. min^−1^) was significantly different between the two groups (*p*<0.01) such that BQ’s had a mean (standard deviation) 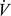
O_2max_ about 11(2.5) mL O_2_. kg^−1^. min^−1^ greater than that of non-BQ’s. There was a non-significant trend (*p* = 0.08) for body mass to differ between groups. During the graded exercise test, BQ’s attained a maximal treadmill speed 20% faster than non-qualifiers (*p*<0.05). Additionally, BQ’s completed the marathon at a higher percentage of v
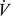
O_2max_ (75±5%) than did non-BQ’s (67±4%, p<0.001). For both groups combined, there was a significant bivariate relation between 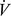
O_2max_ (mL O_2_. kg^−1^. min^−1^) and marathon finishing time (adjusted r^2^ = 0.47, SEE = 37.5 min, *p*<0.05).

Percent of predicted values for a lung function tests were not significantly different between groups (Table 2). Three subjects in each group had a DLNO greater than the upper limit of normal (ULN). Two BQ’s had a DLCO above the ULN, while 5 non-BQ’s had a DLCO that surpassed the ULN. Chi-square analysis reveals that there is not a significant difference in the proportion of subjects with an abnormally high diffusion capacity (DLCO or DLNO) between groups (data not included).

**Table 2:** Pulmonary function measurements for Boston Qualifiers and non-Qualifiers.

	BQ (n = 10)		Non-BQ (n = 18)		Total (n = 28)	
	Mean	Percent Predicted	Mean	Percent Predicted	Mean	Percent Predicted
FVC (L)	5.1 (0.9)	110 (8)*	5.0 (1.0)	105 (14)	5.0 (0.9)	106 (13)*
	3.8–6.4	96–124	3.4–7.2	79–134	3.4–7.2	79–134
FEV_1_ (L)	4.0 (0.7)	108 (11)*	3.8 (0.7)	100 (11)	3.9 (0.7)	103 (11)
	2.9–5.4	96–128	2.5–5.1	86–129	2.5–5.4	86–129
FEV_1_/FVC	81.2 (10.6)	99 (8)	77.2 (6.3)	96 (9)	78.6 (6.2)	97 (8)
	73.4–90.2	89–113	64.9–87.2	77–110	64.9–90.2	77–113
PEF (L)	9.0 (2.2)	102 (11)	10.1 (2.0)	111 (10)*	9.7 (2.1)	108 (11)*
	6.0–12.9	81–118	7.0–13.1	97–137	6.0–13.1	81–137
FEF_25–75_ (L/s)	5.2(1.5)	133 (32)*	4.8 (1.3)	129 (29)*	5.0 (1.3)	131 (29)*
	3.2–8.3	91–192	2.7–6.8	84–176	2.7–8.3	84–192
DLCO	34.7 (5.9)	113 (15)*	34.3 (6.6)	114 (14)*	34.3 (6.73)	113 (14)*
	25.9–43.4	91–135	22.6–48.6	93–146	22.6–48.6	91–146
DLCO/BSA	20.0 (3.1)	–	18.3 (2.6)	–	18.9 (2.8)	–
	15.5–23.9	–	13.6–23.6	–	13.6–23.9	–
DLNO	179 (27)	113 (12)*	176 (34)	113 (13)*	175 (35)	113 (13)*
	140–212	97–130	124–256	94–149	120–256	94–149
DLNO/BSA	103 (14)	–	93 (13)	–	97 (14)	–
	85–123	–	72–124	–	72–124	–
V_C_ (mL)	90 (15)	116 (19)*	89 (13)	117 (15)*	90 (14)	117 (16)*
	72–113	95–151	64–118	95–144	64–118	95–151

FVC: forced vital capacity; FEV_1_: forced expiratory volume within 1 sec; FEV_1_/FVC: fraction of inspired air expired within 1 sec; PEF: peak expiratory flow; FEF_25–75_: forced expiratory flow during 25–75% of 6-second exhale; DLCO: pulmonary diffusing capacity for carbon monoxide, in mL/min/mmHg; DLCO/BSA: DLCO relative to body surface area, in mL/min/mmHg/m^2^; DLNO: pulmonary diffusing capacity for nitric oxide, in mL/min/mmHg; DLNO/BSA: DLNO relative to body surface area, in mL/min/mmHg/m^2^; V_C_: pulmonary capillary blood volume.

Data are reported as mean (SD) values and range.

* Denotes a significant difference in observed parameter relative to predicted (p<0.05).

Mean finishing time for all subjects was 220.0 minutes, (range = 142.6 to 288.9). Weight loss from the marathon was comparable between the two groups [1.0 (1.4) kg for BQ’s; 0.9 (0.8) kg for non-BQ’s, p = 0.746]. Ten of the twenty-eight participants (6 males, 4 females) qualified for the Boston Marathon (average time = 180.0+23.1 min); the average time for the remaining 18 (12 males, 6 females) was 242.2+28.3 minutes. Finishing time was significantly faster in BQ’s when controlling for age and gender (*p*<0.01). Step-wise linear regression determined that finishing time for all subjects was dependent on maximum treadmill speed and specific endurance (adjusted r^2^ = 0.97, SEE = 6.9 minutes *p*<0.05); however, 80% of the variance in finishing time is accounted for by differences in maximum treadmill speed sustained for 60 seconds alone (adjusted r^2^ = 0.80, SEE = 17.7 minutes, *p*<0.05).

**Figure 1:**
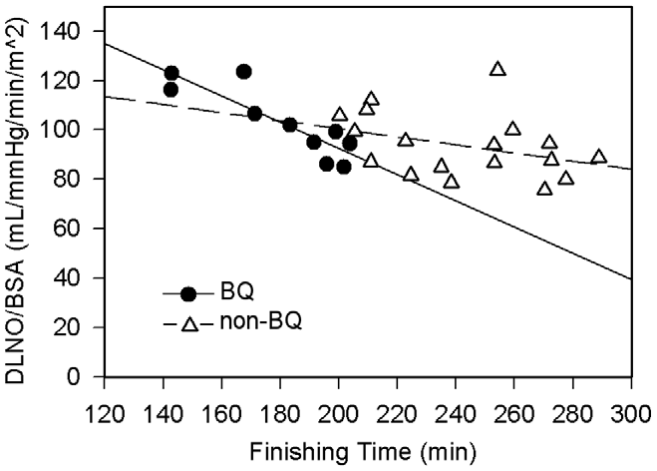
Regression showing relation between DLNO/BSA and marathon finishing time. For Boston Qualifiers (solid line), r^2^ = 0.74, SEE = 11.8, *p*<0.01, showing a significant correlation. Y-intercept is 198.8+18.7 (95% confidence interval ranges from 155.7 to 242.0); slope of the line is −0.532+0.103, with a 95% confidence interval range of −0.8 to −0.3. For non-qualifiers (dashed line), *p* = 0.14. Y-intercept is 135.7+26.5 (95% confidence interval ranges from 79.5 to 191.8); the slope of the line is −0.175+0.109, with a confidence interval range of −0.4 to 0.05. The point of intersection for these lines is 178.07 minutes (2∶58:11). There is a significant difference between the correlation coefficients of the two regressions (2-tailed *z* = −2.15, *p* = 0.03).

**Figure 2:**
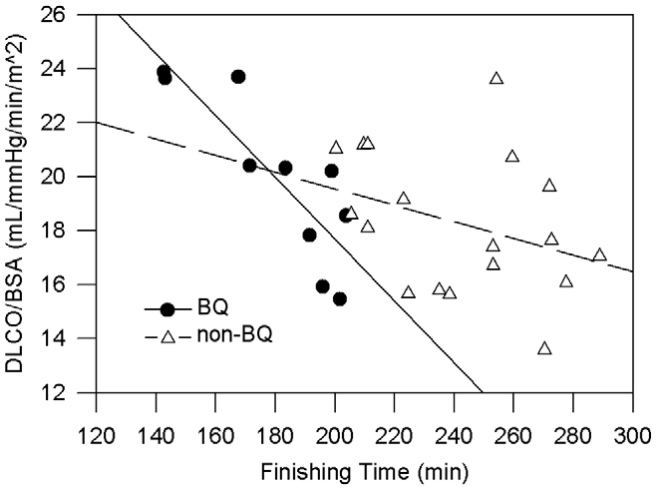
Regression showing relation between DLCO/BSA and marathon finishing time. For Boston Qualifiers (solid line), r^2^ = 0.69, SEE = 12.8, *p*<0.01, showing a significant correlation. Y-intercept of the line is 40.6+4.5, with a 95% confidence interval range of 30.2 to 51.0. The slope of the line is −0.11+0.03, with a confidence interval of −0.18 to −0.06. For non-qualifiers (dashed line), *p* = 0.172. Y-intercept is 25.7+5.2, confidence interval ranges from 14.6 to 36.8. The slope of the line is −0.03+0.02, with a 95% confidence interval ranging from −0.08 to 0.02. The point of intersection for these lines is 179.22 minutes (2∶59:14). The correlation coefficients of these lines are significantly different (2-tailed *z* = −1.99, *p* = 0.046).

In BQ’s, the strongest relation identified was between finishing time and DLNO normalized to BSA. For non-BQ’s, finishing time was best predicted by maximum treadmill velocity sustained for 60 seconds and specific endurance, where 74% of the variance in finishing time is accounted for by differences in maximum treadmill velocity. The relation between DLNO normalized to BSA and finishing time was not significant for non-BQ’s (*p* = 0.127). A significant difference was found between the correlation coefficients of the two linear regressions (two-tailed *z* = −2.15, *p* = 0.03). When regression lines of DLNO normalized to BSA versus finishing time plotted on the same axes, the regressions intersect at a point corresponding to a finishing time of 178.1 minutes (Figure 1), suggesting that the relation between DLNO normalized to BSA and marathon time begins to change around 237 m. min^−1^ pace (6 minutes, 47 seconds per mile). DLCO normalized to BSA was also correlated to finishing time in BQ subjects, to a lesser extent. For non-BQ, no significant relation between the variables is evident (*p* = 0.46). These regressions intersect at the point corresponding to 184.3 minutes (Figure 2).

## Discussion

The novelty of this study lies in that it shows that the relation between pulmonary factors (measured at rest) and marathon performance may differ between athletes of different skill level. In particular, this study was able to isolate an approximate time point at which the relation between pulmonary diffusing capacity for nitric oxide and marathon finishing time changes for trained endurance athletes, pinpointing a pace at which lung function becomes limiting to performance. Whereas runners qualifying for the Boston Marathon, because of their overall faster pace, are limited by DLNO, non-qualifiers probably experience a more mechanical limitation, such as leg turnover (related to maximum treadmill velocity).

The primary purpose of this study was to compare the correlation of DLNO and DLCO to marathon running performance. The results demonstrate that there was a significant slope (indicating a strong correlation) between DLNO and DLCO (normalized to BSA) versus marathon finishing time only in runners that qualified for the Boston Marathon, with these variables showing a stronger predictive relation to finishing time than either
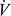
O_2max_ or running economy. DLNO was shown to be the strongest predictor of finishing time, such that every 1 mL. min^−1^. mmHg^−1^.m^−2^ increase in DLNO at rest projects that finishing time will decrease by about 1.4 minutes (with a range of 0.8 to 2.1 min). These results strongly suggest that alveolar-capillary membrane conductance may be performance-limiting in runners that complete a marathon in 3 hours or faster, as shown by the intersection of regression lines for BQ and non-BQ groups (Figure 1 and 2). These figures also demonstrate that DLNO relative to BSA is a more accurate predictor of finishing time than DLCO, as the former correlation shows a larger adjusted r^2^ and a lower standard error of the estimate.

The physiological mechanism closely relating DLNO to marathon performance in BQ’s is speculative, given that these subjects ran at approximately 75% of 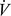
O_2max_, a value consistent elsewhere in those with similar running abilities [10], [13]. Though arterial oxygen pressure and the alveolar-to-arterial oxygen pressure difference was not measured throughout the race in this study, others have shown hypoxemia is not induced in fit athletes running half of a full marathon at ∼75% 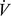
O_2max_ [36]. Nonetheless, we suggest that, in well-trained runners, there is a 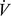
O_2_ threshold at which pulmonary diffusion limits oxygen consumption. In other words, these individuals run the marathon at a speed at which 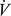
O_2_ is high enough that gas diffusion at the alveolar-capillary membrane becomes a physiological bottleneck, and those with greater alveolar-capillary membrane conductance are able to maintain greater arterial oxygen saturation. Similarly, non-BQ’s likely complete the marathon at a 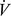
O_2_ at which pulmonary diffusion is not limiting; this may explain the lack of relation between DLNO and performance in this group. Thus, individuals who have superior alveolar-capillary membrane conductance (high DLNO measurements), and yet do not reach a “heart” or “muscle limitation,” (i.e., non-BQ’s) would not have any performance advantage over other individuals who have lower alveolar-capillary membrane conductance. In fact, studies have demonstrated that marathon running causes a significant drop in pulmonary diffusing capacity [29], [37]. About 30% of the drop in DLNO (normalized to BSA) post-exercise is accounted for by marathon finishing time (*p* = 0.046) [29]. Thus, for every 1 minute improvement in marathon time, DLNO is reduced by 1.2 mL.min^−1^.mmHg^−1^.m^−2^ [29]. The diminished DLNO with marathon running can be expected, since 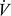
O_2max_ accounts for about 40 to 77% of the variance in marathon performance [5], [8], [9], [10], [11] and about 40% of the change in DLNO from pre- to post-exercise [38]. Therefore, it is possible that individuals with larger alveolar-capillary membrane conductance at the start of the marathon have a physiological advantage: diffusion impairments that may arise during the race will likely not decrease diffusion capacity to problematic levels.

Only one other study to date has examined the association between pulmonary diffusion and marathon finishing time using both DLNO and DLCO as predictors [29]. While Manier and colleagues did not intend to examine this association, the data set was available in the publication. In BQ runners (n = 9, mean finishing time  = 177.0±15.0 min), the relation between DLNO indexed to BSA and finishing time was present, but it was not as strong as in the current study (adjusted r^2^ = 0.30, SEE = 12.5 min, *p* = 0.073). In Manier’s study, for every 1 mL.min^−1^.mmHg^−1^.m^−2^ increase in DLNO at rest, marathon finishing time was 0.8 minutes faster (ranging from 1.8 minutes faster to 0.1 minute slower) [29]. Combining these data with those from the present study suggests that 30 to 74% of the variance in DLNO (mL. min^−1^. mmHg^−1^.m^−2^) at rest is related to marathon finishing time in BQ’s, while no such relation exists in non-BQ’s. Additionally, controlling for age and gender does not affect this correlation in either study separately or collectively [29]. Combining the data from these independent studies further supports that pulmonary diffusing capacity is an important contributor to marathon performance in well-trained runners. It is also important to note that while the BQ group represents a well-trained population and some of the subjects in this study performed at a very high level, international class runners tend to have even higher values of 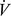
O_2max_ and, possibly, an even greater dependence on alveolar-capillary membrane conductance.

Although DLNO normalized to BSA was only related to finishing time in BQ’s, DLNO did not significantly differ between BQ and non-BQ whether indexed to BSA (p = 0.078) or not (p = 0.80). DLNO is usually higher in fit subjects [21] and in the present study, DLNO was significantly higher than predicted whether using norms from Zavorsky and colleagues (113% predicted, p<0.01) [34] or Aguilaniu and colleagues (107% predicted, p = 0.011) [39]. Several subjects in both BQ and non-BQ groups had values above the upper limit of normative data for a variety of pulmonary function parameters. As such, we can conclude that BQ and non-BQ were of comparable respiratory fitness. These findings suggest that endurance training itself may improve alveolar-capillary membrane conductance above that of untrained individuals, but improvements in DLNO likely plateau well before that of the heart or skeletal muscle.

Generally speaking, the lungs become limiting at a running pace of 6∶47 minutes per mile or 236 m.min^−1^ (a 3 hour marathon). Therefore, athletes performing at or around this pace should be aware of the potential significance of this limitation and its ramifications for performance. It is unknown whether any specific training practices can be implemented to improve pulmonary diffusion and therefore improve marathon performance.

This study is limited by its small sample size; a higher power to detect differences in group means would likely be achieved by recruiting more participants. Nevertheless, it is frowned upon to conduct a *post-hoc* power analysis after data collection has occurred [40], [41], thus we did not perform one. Instead, confidence intervals replace power calculations after a study is completed [40], [41]. We have provided confidence intervals in Figures 1 and 2. Additionally, small group sizes result in large variances; as such, the standard errors of the estimate for both regressions are large and overlapping, obscuring estimation of a clear range of intersection at which DLNO indexed to BSA begins to predict finishing time. Larger sample sizes would also allow us to divide runners into more specific categories by time, possibly delineating a clearer relation between DLNO indexed to BSA and finishing time with increasing running speed. Additionally, normalization to BSA transforms DLNO into a variable with multiple units, possibly complicating analysis. Mean height and body mass themselves were not significantly different between groups; however, the combination of these variables appears to be important when related to pulmonary variables. It remains possible that BSA introduces variation in the data set due to its relationship with heat dissipation, largely dependent on stature [42]. This might have impacted finishing time, especially in slower runners finishing as the ambient temperature increased on race day, from about 8°C (96% humidity) at the 8∶00 AM start to 21°C (51% humidity) at the finish line by 1∶00 PM.

As pulmonary diffusion has been shown to decrease during long-duration submaximal exercise [29], [37], the efficacy of interventions which may counter these negative effects should be explored (e.g., anti-inflammatory drugs or antioxidants). Our results could be strengthened by measuring DLNO immediately after completion of the marathon, with a greater change in DLNO representative of presence of a limitation. Nevertheless, measurement of DLNO at rest may still underestimate severity of diffusion limitation. As such, future studies could also measure DLNO at submaximal speeds during running economy testing and extrapolate these data to marathon race pace, allowing a more accurate estimation of the impact of diffusing capacity on running performance.

The genetic basis of DLNO could be further studied to determine how this parameter might change as one adapts to training. Furthermore, longitudinal and interventional studies are recommended to determine if any specific type of training can optimize pulmonary diffusion capacity and therefore improve endurance running performance. More extensive understanding of the relationship outlined in this study will allow us to confirm the validity of diffusing capacity for nitric oxide as a fitness predictor.

In conclusion, this study found that DLNO indexed to BSA is a better predictor of marathon finishing time in runners qualifying for the Boston Marathon than are more commonly used variables, such as 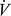
O_2max_ or running economy, but this relation was not observed for non-BQ’s. This suggests that alveolar-capillary membrane conductance can be pulmonary limitation in well-trained runners.
